# Diversity of RNA viruses of three dominant tick species in North China

**DOI:** 10.3389/fvets.2022.1057977

**Published:** 2023-01-13

**Authors:** Tong Qin, Mingjie Shi, Meina Zhang, Zhitong Liu, Hao Feng, Yi Sun

**Affiliations:** ^1^State Key Laboratory of Pathogen and Biosecurity, Beijing Institute of Microbiology and Epidemiology, Academy of Military Medical Sciences, Beijing, China; ^2^Medical Corps, Naval Logistics Academy, PLA, Beijing, China

**Keywords:** virome, diversity, high-throughput sequencing, Ixodidae, China

## Abstract

**Background:**

A wide range of bacterial pathogens have been identified in ticks, yet the diversity of viruses in ticks is largely unexplored.

**Methods:**

Here, we used metagenomic sequencing to characterize the diverse viromes in three principal tick species associated with pathogens, *Haemaphysalis concinna, Dermacentor silvarum*, and *Ixodes persulcatus*, in North China.

**Results:**

A total of 28 RNA viruses were identified and belonged to more than 12 viral families, including single-stranded positive-sense RNA viruses (*Flaviviridae, Picornaviridae, Luteoviridae, Solemoviridae*, and *Tetraviridae*), negative-sense RNA viruses (*Mononegavirales, Bunyavirales*, and others) and double-stranded RNA viruses (*Totiviridae* and *Partitiviridae*). Of these, Dermacentor pestivirus-likevirus, Chimay-like rhabdovirus, taiga tick nigecruvirus, and Mukawa virus are presented as novel viral species, while Nuomin virus, Scapularis ixovirus, Sara tick-borne phlebovirus, Tacheng uukuvirus, and Beiji orthonairovirus had been established as human pathogens with undetermined natural circulation and pathogenicity. Other viruses include Norway mononegavirus 1, Jilin partitivirus, tick-borne tetravirus, Pico-like virus, Luteo-like virus 2, Luteo-likevirus 3, Vovk virus, Levivirus, Toti-like virus, and Solemo-like virus as well as others with unknown pathogenicity to humans and wild animals.

**Conclusion:**

In conclusion, extensive virus diversity frequently occurs in *Mononegavirales* and *Bunyavirales* among the three tick species. Comparatively, *I. persulcatus* ticks had been demonstrated as such a kind of host with a significantly higher diversity of viral species than those of *H. concinna* and *D. silvarum* ticks. Our analysis supported that ticks are reservoirs for a wide range of viruses and suggested that the discovery and characterization of tick-borne viruses would have implications for viral taxonomy and provide insights into tick-transmitted viral zoonotic diseases.

## Introduction

Ticks (Arachnida: Ixodida) are common hematophagous arthropods that have been implicated as vectors of human and animal diseases worldwide (1). Approximately 900 species of ticks have been described and taxonomically classified into four families *Argasidae* (soft ticks), *Ixodidae* (hard ticks), *Nuttalliae*, and the newly proposed *Deinotheriidae* (2, 3). Their propensity for feeding on multiple hosts, expansive range, and long life cycle underlines the importance of active surveillance of ticks for the presence of potential pathogens threatening both human and animal health (4). Argasid and ixodid ticks combined transmit a greater diversity of viral, bacterial, and protozoan pathogens than any other arthropod vectors (5). The increasing incidence of tick-borne disease worldwide is caused partly by the increased frequency of human exposure to ticks or their endemic habitats, burgeoning tick populations, and the discoveries of new tick-associated agents (5, 6). The significance of tick-borne viral diseases (TBVDs) in human health has raised global health concerns in recent decades due to current suboptimal diagnostics, treatment options for emerging viruses, and a scarcity of vaccines (7). Attention from clinicians and researchers had alerted the public to the frequent emergences of novel viral pathogens causing febrile human diseases, such as Dabie bandavirus, also known as severe fever with thrombocytopenia syndrome virus (SFTSV) (8, 9), Alongshan virus (10), Bourbon virus (11), most recently Jingmen tick virus (12), Yezo virus (13), Toyo virus (14), and Songlin virus (15), which had been identified in China and neighboring countries. In addition, the re-emergence and continuous spread of known tick-borne viral diseases (TBVDs), such as Crimean–Congo hemorrhagic fever virus (CCHFV) (16), Heartland virus (17), tick-borne encephalitis virus (18), Powassan virus (19), and African swine fever virus (20), positively correlate with the increasing incidence of TBVDs in humans and animals. The emergence of some undefined TBVDs pathogens as well as the dearth of data on tick virome highlights an urgent requirement for active viral surveillance and discovery in ticks, which greatly promotes our knowledge of the biodiversity and evolution of viruses transmitted by ticks, although it seems plausible that traditional isolation *via* tissue culture in cell lines has been proven a prerequisite gold standard to characterize a novel virus or insight into a known one (5). However, not all tick-borne viruses are amenable to being isolated due to technological limitations, extensive culture-independent studies, such as transcriptomics analysis following the high-throughput sequences (HTSs), have been attempted to examine tick virome (21, 22). Such studies might not only identify viruses associated with acute diseases but also could provide insights into the pathogenesis of more controversial chronic illnesses associated with tick bites (23). Therefore, profiling viruses in ticks and investigating the substantial contact between ticks and human or animal hosts are necessary to better understand the viral sphere harbored by ticks and identify the potential viral pathogens in China. In this study, the virome of tick pools was profiled *via* metagenomic sequencing aimed at identifying the baseline viruses carried by ticks and characterizing their biodiversity and evolution. Then, the viral transmission patterns between ticks and humans were discussed based on the active surveillance results of molecular prevalence and potential exposure of humans to these ticks. These findings may shed light on the tick virome endemic to China and reveal the potential risks of TBVDs in the human population, which helps to guide tick-borne disease prevention and control in China.

## Materials and methods

### Tick collection, visual assignment, and DNA/RNA preparation

Questing *Ixodes persulcatus, Haemaphysalis concinna*, and *Dermacentor silvarum* were collected by sweeping flags on the vegetation in Zhalantun county, Inner Mongolia Autonomous Region, and Mudanjiang city, Heilongjiang province, respectively (Supplementary Figure 1). Morphological identification of ticks was conducted using the standard key for Chinese ticks (24) under stereomicroscopes. We further assigned these ticks into eight pools by species, sex/nymph, and origin location, each with 30 individuals (the nymphal pool of *H. concinna* was abandoned for inadequate individuals). All tick specimens were thoroughly surface sterilized to remove the possible contaminants before DNA/RNA isolation. Surface sterilization included sequential 1 mL washes with a vortex for 1 min in 3% hydrogen peroxide (H_2_O_2_), two 30 s washes in 70% ethanol (w/v), 2 min in phosphate-buffered saline (PBS, pH = 7.4), and finally drying on clean tissue paper to avoid cross contaminations. Extraction of total DNA and RNA from ticks was performed using E.Z.N.A. DNA/RNA Isolation Kit (OMEGA) with modifications. In brief, ticks were homogenized in RLT solution under liquid nitrogen and then incubated at 55°C for 10 min with proteinase K (Qiagen) and centrifuged for 30 s at 15,000 × *g*. We took a proportion (~30 μL) of the homogenates for genomic DNA extraction with RNAase A treatment (15 min) for later DNA purification. Simultaneously, another 300 μL of tick homogenates was utilized to enrich viral particles and total RNA extraction. Before RNA extraction, the homogenates were filtered (0.45 μM) at room temperature and then treated with RNase A (15 min), followed by Turbo DNase and Benzonase (MilliporeSigma, Burlington, MA, USA) (30 min) (22). The enrichment of viral particles had been achieved due to the degradations of unprotected nucleic acid absence of a viral capsid (25).

### Validation of tick identifications

To verify tick species identification, genomic DNA was extracted from each specimen pool using E.Z.N.A. DNA/RNA Isolation Kit (OMEGA). Two genes were used for tick identification: the partial 18S rRNA gene (~1,100 nt) which was amplified using primer pairs 18S-1 (5′-CTGGTGCCAGCGAGCCGCGGYAA-3′) and 18S-2 (5′-TCCGTCAATTYCTTTAAGTT-3′) and partial COI gene (~680 nt) using primer pairs LCO1490 (5′-GGTCAACAAATCATAAAGATA TTGG-3′) and HCO2198 (5′-TAAACTTCAGGGTGACCAAAAAATCA-3′). PCRs were performed as previously described (26, 27). For taxonomic determination, the resulting sequences were compared against the non-redundant nucleotide (nt) database and with all COI barcode records on the Barcode of Life Data (BOLD) Systems.

### Library preparation and sequencing

The enriched total nucleic acid (11 μL) from each tick pool was then subjected to first- and second-strand cDNA synthesis with Super Script IV reverse transcriptase (Invitrogen, Waltham, MA, USA) and exo-Klenow fragment (New England Biolabs, Ipswich, MA, USA), respectively. For all libraries, the Ribo-Zero Gold Kit (Illumina) was used to remove ribosomal RNA under the manufacturer's guidance. Subsequently, all rRNA-depleted RNA samples were resuspended to construct libraries using the KAPA Stranded RNA-Seq Kit (KAPA biosystems, Roche) with TruSeq Index PCR Primer barcodes (Illumina, SanDiego, CA, USA) following the manufacturer's instructions. Qubit high-sensitive RNA assays (Thermo Fisher Scientific) were performed to quantify cDNA levels before, during, and after library preparation, and the fragment sizes were simultaneously determined with an Agilent Bioanalyzer. Subsequently, equimolar amounts of nucleic acids were pooled and submitted for sequencing in each library. All libraries were sequenced on a single lane (paired-end, 125 bp read-length) on an Illumina HiSeq 2,500 platform at the BGI Sequencing Center (www.genomics.cn).

### Quality checking, trimming, and *de novo* assembly

Raw sequencing reads were first subjected to adapter removal and quality trimmed with TrimGalore (www.bioinformatics.babraham.ac.uk/projects/trim_galore/) (28, 29). Clean reads were *de novo* assembled using the Trinity v2.8.5 program (30, 31).

### Virus discovery and genome annotation

Trinity assemblies with a length above 200 bp were subjected to BLASTN against all nt databases using a local BLAST tool and BLASTX against all non-redundant protein (nr) databases (available as early as May 2022) of reference RNA viruses as well as those recently published, with hits at an e-value of 1 × 10^−5^ or better collated. All potential virus assemblies were screened against the Conserved Doman Database (www.ncbi.nlm.nih.gov/Structure/cdd/wrpsb.cgi) with an expected value threshold of 1 × 10^−3^ to identify viral gene segments. Putative viral contigs were further merged by high-identity overlaps with a threshold value of 95% similarity using the SeqMan program of the Lasergene package v7.1 (32). To complete the remaining gaps, original reads were aligned to the viral contigs again using the Bowtie2 program (33), and the resultant assembly was verified in the Integrated Genomics Viewer (34). A novel viral species should be satisfied with one of the following conditions described before (35), namely (i) <80% nucleotides identity across the complete genome or (ii) <90% amino acids identity of RNA-dependent RNA polymerase (RdRp) domain with the known viruses. To eliminate possible endogenous viruses, all viral assemblies were blasted against the reference genomes of *I. persulcatus* (GCA_013339685.1), *D. silvarum* (GCA_013339745.1), and whole-genome shotgun database of Ixodida (Taxonomy ID: 6935, accession data:1/1/2022), respectively. If aligned bases of the query contigs covered more than 50% and the nucleotide similarity exhibited higher than 85% from any comparison with the earlier databases, they were discarded from the downstream analysis. Transcript abundance containing RNA-Seq fragment counts for each transcript (or gene) across each sample was estimated using the alignment-based abundance estimation method RSEM (36). The trimmed mean of M-values normalization method (TMM) was employed to normalize the transcript abundance.

### Viral PCR confirmation and full-length genome walking

Reverse transcription-PCR and nested PCR were performed to confirm the existence of virus on RNA samples from the tick pools which were originally subjected to RNA-seq with primer sets (Supplementary Table 2) targeting the RdRp contigs of virus hit. Then, these primers were utilized to really exclude the presence of endogenous viral elements by PCR amplifications on genomic DNA templates from the same tick pools. Only positively detected potential virus would undergo a further epidemiological survey. The contigs which hit the viral genome were used as templates for overlapping primers design. The overlapping primers for amplifying the complete genome of the representative viruses (Taiga tick Nigecruvirus and Mukawa virus) were provided for the nested RT-PCR, genome walking, and rapid amplification of complementary DNA ends (5′-, 3′-RACE) with a commercial kit (Takara) according to the manufacturer's protocol (Supplementary Tables 3, 4). All the PCR products were separated on 0.8% agarose gel and purified through an agarose gel DNA extraction kit for Sanger sequencing.

### Multiple sequence alignments and evolutionary analysis

To infer the evolutionary relationships of the viruses discovered, the protein-translated RdRp open reading frame segments produced in this study were combined with representative complete proteomes and (or) RdRp-segments of the *Bunyavirales, Mononegavirales, Flaviviridae, Luteoviridae, Partitiviridae, Totiviridae*, and *Picornaviridae* were retrieved from NCBI GenBank (www.ncbi.nlm.nih.gov/genbank) and aligned using MAFFT v.7.266, employing the E-INS-i algorithm (37). Ambiguous regions in the alignments were removed with TrimAl v.1.2 (38). Following sequence alignment, ProtTest v.3.4 was employed to select the best-fit model of amino acid substitution (39). Finally, maximum likelihood trees for all alignments were inferred using the best-fit model of amino acid substitution (LG + I + Γ + F for all alignments) with 1,000 bootstrap replicates with the PhyML v.3 program (40). Phylogenetic trees were edited and visualized with FigTree v.1.4.2 (http://tree.bio.ed.ac.uk/software/figtree). All phylogenetic trees were mid-point rooted for purposes of clarity only. The flowchart of the study design diagram was illustrated with different types of treatment and analysis (Supplementary Figure 2).

## Results

After morphological classification, there were 216 *I. persulcatus* (129 females, 52 males, and 35 nymphs) and 147 *H. concinna* (87 females, 49 males, and 11 nymphs) achieved alive from Mudanjiang city, Heilongjiang province, which were then randomly assigned into five groups of 30 individuals each species, sexes, and development stage. Among them, *I. persulcatus* groups included female, male, and nymphal ones, respectively, while only two groups (female and male) of *H. concinna* were arranged for inadequate nymphs. An average of 10.96 Gb data, including 7.1–7.6 × 10^7^ or so 150-base paired-end reads, were generated from male, female, and nymph groups of *I. persulcatus*, while the male and female groups of *H. concinna* generated ~11.18 Gb of data on average, including 6.8 × 10^7^ and 8.0 × 10^7^ or so 150-base paired-end reads. While in Zhalantun city, Hulunbuir, Inner Mongolia, a total of 274 *D. silvarum* (58 females, 170 males, and 46 nymphs) and 40 *I. persulcatus* (28 females and 12 males) were harvested alive. The *D. silvarum* ticks were also divided into three groups (female, male, and nymphal ones), each with 30 individuals, which generated ~12.30 Gb data on average. After quality filtration and host subtraction, a total of 614,213,814 reads remained which were assembled into 815,457 contigs. Assembled contigs were compared to the NCBI Viral Genomes database, resulting in 1,311, 1,033, and 709 viral contigs from *I. persulcatus, D. silvarum*, and *H. concinna* as viral origin through BLASTN and BLASTX, which were kept for a subsequent manual inspection (Supplementary Table 1). A prediction of ORFs was also implemented and compared to the viral protein database through BLASTP. As a result, sequences of 28 putative viruses were identified (Table 1), with five representing novel species. Seventeen viruses were identified within the *I. persulcatus* pool, 11 in *D. silvarum*, while only six were discovered in *H. concinna*. Of them, single-stranded positive-sense RNA viruses (*Flaviviridae, Luteoviridae, Picornaviridae, Solemoviridae*, and *Tetraviridae*), negative-stranded RNA viruses (*Mononegavirales* and *Bunyavirales*), and double-stranded RNA viruses (*Totiviridae* and *Partitiviridae*) were involved (Table 1). The presence of the representative viral strains in the corresponding pool was further verified by nested RT-PCR and Sanger sequencing (Supplementary Tables 2, 3, Supplementary Figures 3, 4). In total, 41 viral sequences from the 28 identified RNA viruses were verified and submitted to NCBI under the accession numbers OP863261–OP863295 and OP863301–OP863305.

**Table 1:** Virus identified in three dominant tick species in North China.

**Voucher species**	**Genus**	**No. unique contigs**	**contig length**	**Encoded fragments**	**mean seq. depth**	**relative abundance (RdRp%)**	**RdRp Identity**	**Top hit (RdRp accession no)**
*Ixodes persulcatus*	Mivirus	3	10,853	N, L, G, VP4	1,107–3,210	0.01–0.02	99.95	Nuomin virus (UKS70436.1)
	Nigecruvirus	3	11,436	N, L, G, VP4	2,103–3,309	<0.01	83.70	Blacklegged tick Chuvirus 2 (AUW34382.1)
	Nucleorhabdovirus	3	2,033–5,986	L, G	650–1,645	0.01–0.02	88.44	Chimay rhabdovirus (AVM86063.1)
	Alphanemrha virus-like	4	5,717–6,663	L, N	1,905–2,221	0.07	49.46	Norway mononegavirus 1 (ASY03261.1)
	Ixovirus	2	6,650–6,680	L, N	1,105–4,732	0.25–0.47	99.67	Sara tick phlebovirus 1 (QPD01621.1)
	Ixovirus	8	1,380–6,630	L	277–22,219	0.02–0.07	74.1	Scapularis ixovirus (YP_010086238.1)
	*Orthonairovirus*	2	12,840–12,869	L, S	105–4,732	0.01	99.04	Beiji nairovirus (UFP37779.1)
	*Orthonairovirus*	4	7,842–11,076	L, N	1,137–4,326	<0.01	93.25	South Bay virus (ANT80542.1)
	Orthobunyavirus	3	5,780–5,800	L, G	1,237–2,938	<0.01	38.25	Ixodes scapularis bunyavirus (BBD75425.1)
	unclassified Bunyavirales	6	4,295–9,080	L	2,231–3,091	<0.01	46.78	Ubmeje virus (QKK82912.1)
	Deltapartitivir us-like	3	1,510–1,628	L	1–521	0.01–0.02	98.1	Jilin partiti-like virus 1 (QTZ97684.1)
	Luteoviridae	2	1,419–1,628	L	427–469	<0.01–0.07	93.8	Norway luteo-like virus 2 (ASY03255.1)
	Luteoviridae	3	1,398–1,610	L	178–506	<0.01–2.62	84.04	Norway luteo-like virus 3 (ASY03257.1)
	Riboviria	2	5,389–5,427	L, N	1,793–1,797	0.25–0.47	44.24	Vovk virus (QKK82915.1)
	Levivirus	2	620–1,899	L, H4bulk, coat	206–632	<0.01	42.27	Levivirus *sp*. (QDH88856.1)
	Totivirus	2	1,100–1,330	L	509	0.01–0.02	38.17	Hubei toti-like virus 24 (YP_009336908.1)
	Sobemovirus	2	2,621	L	872	0.01–0.02	39.62	Hubei sobemo-like virus 15 (YP_009330030.1)
*Dermacenor silvarum*	pestivirus-like viruses	4	14,481–15,100	L. S	4,827	0.01	90.81	Bole tick virus 4 (QUJ17979.1)
	Alphanemrha virus-like	3	6,610–6,701	L, N	1,241–3,305	<0.01	94.51	Rhipicephalus associated rhabdo-like virus (QYW06859.1)
	Alphanemrha virus-like	2	6,659–6,700	L	1,893–2,215	0.01	48.42	America dog tick rhabdovirus (AUX13124.1)
	Ixovirus	1	6,650	L, S	4,732	0.27	99.41	Sara tick phlebovirus 1 (QPD01621.1)
	Phlebovirus	2	2,890–3,300	L, N, Ns	1,079	0.04	93.53	Mukawa virus (YP_009666332.1)
	Uukuvirus	3	4,537–4,550	L	1,513	0.04	92.61	Tacheng uukuvirus (AWK68110.1)
	*Orthonairovirus*	4	7,842–11,076	L, N	1,137–4,326	<0.01	93.25	South Bay virus (ANT80542.1)
	*Solemoviridae*	2	722–1,440	orf1, orf2	522–1,328	<0.01	100	Xinjiang tick associated virus 1 (QBQ65134.1)
	*Picornavirus*	2	648–684	L	316–328	0.01–0.07	33.79	Xiangshan picorna-like virus 7 (UDL13977.1)
	Totivirus	2	2,008–4,566	L, orf1	774–1,052	0.01	45.10	Lonestar tick totivirus (AUX13136.1)
	Totivirus	3	572–881	L	235	<0.01	98.31	Xinjiang tick totivirus (QBQ65052)
	Unclassified Tetravirus	3	5,310–5,514	L	1,345–1,737	<0.01	71.53	tick borne tetravirus (QTE18640.1)
*Haemaphyaslis concinna*	Alphanemrha virus-like	1	7,238	L	2,170	0.01–0.02	99.69	Tahe rhabdovirus 2 (UXX19014.1)
	Nucleorhabdovirus	4	1,350–1,568	L, N	537	0.01	73.53	blacklegged tick rhabdovirus-1 (AUW34390.1)
	Alphanemrha virus-like	2	3,489–6,500	L	1,133–2,169	0.01–0.02	48.29	Tacheng Tick Virus 3 (YP_009304331.1)
	Alphanemrha virus-like	2	2,227–2,415	L, N	775–804	0.01	51.40	Bole tick virus 2 (YP_009287864.1)
	Sobemovirus	2	3,700–5,092	L	1,228–1,678	0.01	75.65	Hubei sobemo-like virus 15 (YP_009330029.1)
	Totivirus	2	2,008–4,566	L, orf1	774–1,052	0.01	50.01	Lonestar tick totivirus (AUX13136.1)

### SsRNA (+) viruses

**Figure 1:**
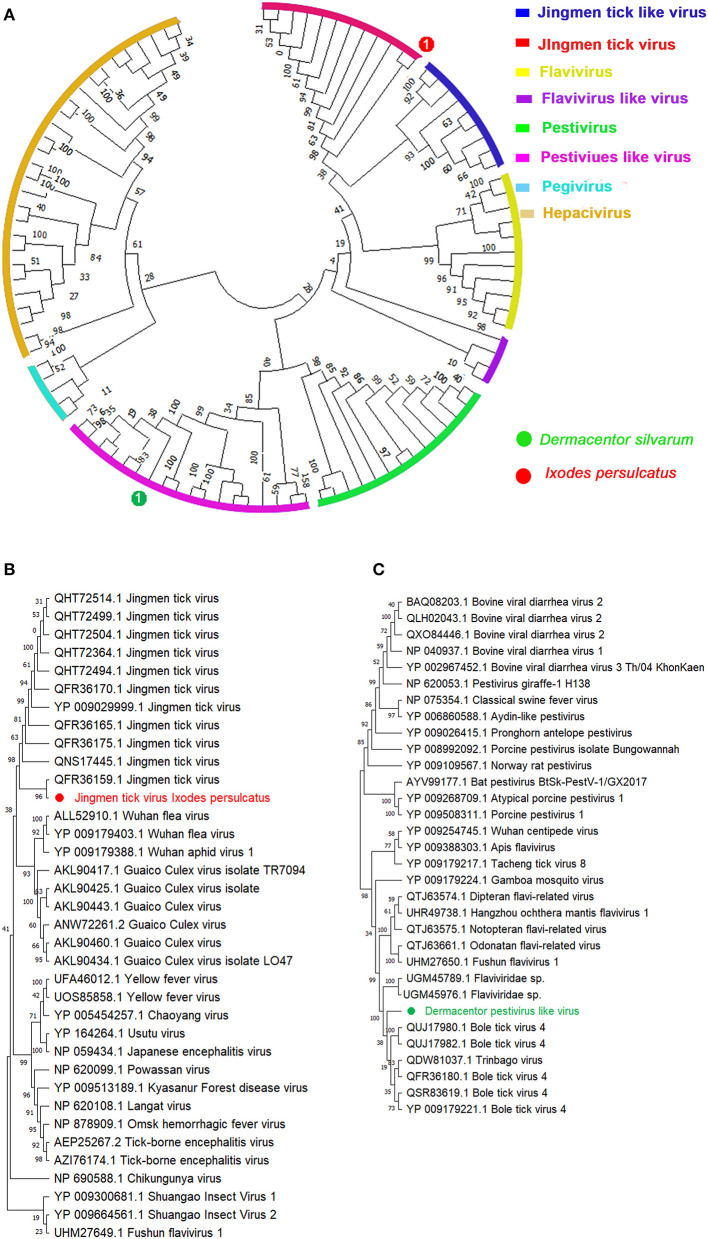
Phylogenetic analysis of representative branches in the family *Flaviviridae* based on the RdRp sequences. **(A)** Enhanced region showing the relationship of the unclassified *Pestivirus*-like group in relation to *Pestivirus* and *Flavivirus*. **(B)** Enhanced region showing the relationship of Jingmen tick virus in relation to the family *Flaviviridae*. **(C)** Enhanced region showing the relationship of Dermacentor pestivirus-like virus in relation to genus *Pestivirus*. Red dot marked with 1 represents Jingmen tick virus from *Ixodes persulcatus*, and green dot marked with 1 represents Dermacentor pestivirus-like virus from *Dermacentor silvarum*. Maximum likelihood tree inferred using the best-fit model of amino acid substitution (LG + I + Γ + F for all alignments) with 1,000 bootstrap replicates.

Sequences for a novel viral species belonging to the family Flaviviridae, tentatively named *Dermacentor silvarum* pestivirus-like virus 1 (DSPV), were identified in all the three *D. silvarum* pools (Table 1, Figures 1A, B). DSPV comprises a single polyprotein that shares the closest homology within the NS3 and NS5 of viruses within the genus *Pestivirus* (41), which clustered DSPV with other recently identified *Pestivirus*-like viruses, Bole tick virus 4 and Trinbago virus (42). Additional segmented *Flavivirus*, Jingmen tick virus, was also discovered in *I. persulcatus* with 97.16 and 96.90% amino acid similarities with RdRp (QFR36159.1) and VP3 protein (QHW66956.1), respectively (Table 1). These contigs were shown positioned on corresponding sites in our phylogenetic tree of these Flavivirus-associated viruses (43) constructed based on their RdRp genes (Figures 1A, C).

#### Luteovirus, Solemovirus, Picornavirus, and Tetravirus

*Luteovirus, Picornavirus, Tetravirus*, and *Solemovirus* are single-stranded positive-sense RNA viruses that are endowed with important and potential viral pathogen parasites on humans and animals (44). In the present study, taiga tick luteo-like virus was identified with shared amino acid sequences of Norway luteo-virus 2 (93.80%, ASY03255.1) and Norway luteo-virus 3 (84.04%, ASY03257.1), respectively, across 99% RdRp gene (Table 1). The two luteo-like viruses grouped with *Ixodes scapularis*-associated viruses in our ML phylogeny tree, although low abundant Norway luteo-virus 2 was present in one library (Supplementary Figure 7). Moreover, a total of three Solemoviruses were discovered in the present study as well (Supplementary Figure S8). Of which, a novel *Solemoviridae* member, Xinjiang tick-associated virus 1 from *D. silvarum*, was shown 100% identical to those sequences on orf1 and orf2, respectively (Table 1). Sobemovirus-like viruses from *H. concinna* and *I. persulcatus* were found to be identical to Hubei sobemo-like virus 15 (YP_009330030.1) with 75.65 and 39.62% identities on RdRp coverage over 2,620 amino acid sequences, respectively (Table 1). In addition, another positive-sense ssRNA viruses, tick-borne tetravirus and Picorna-like virus from *D. silvarum*, exhibited 71.53 and 33.79% amino acid identities of RNA polymerase genes with tick-borne tetravirus-like virus (QTE18640.1) (45) (Table 1, Supplementary Figure 9) and Xiangshan picorna-like virus 7 (UDL13977.1) over 95% coverage spaced across L segments, respectively (Table 1, Supplementary Figure 10). Further classification of the two ss (+) RNA viruses was challenging due to high sequence divergence in combination with a limited number of available *Tetravirus* and *Picornavirus* sequences.

### SsRNA (-) viruses

#### Mononegavirales

##### Chuviridae

**Figure 2:**
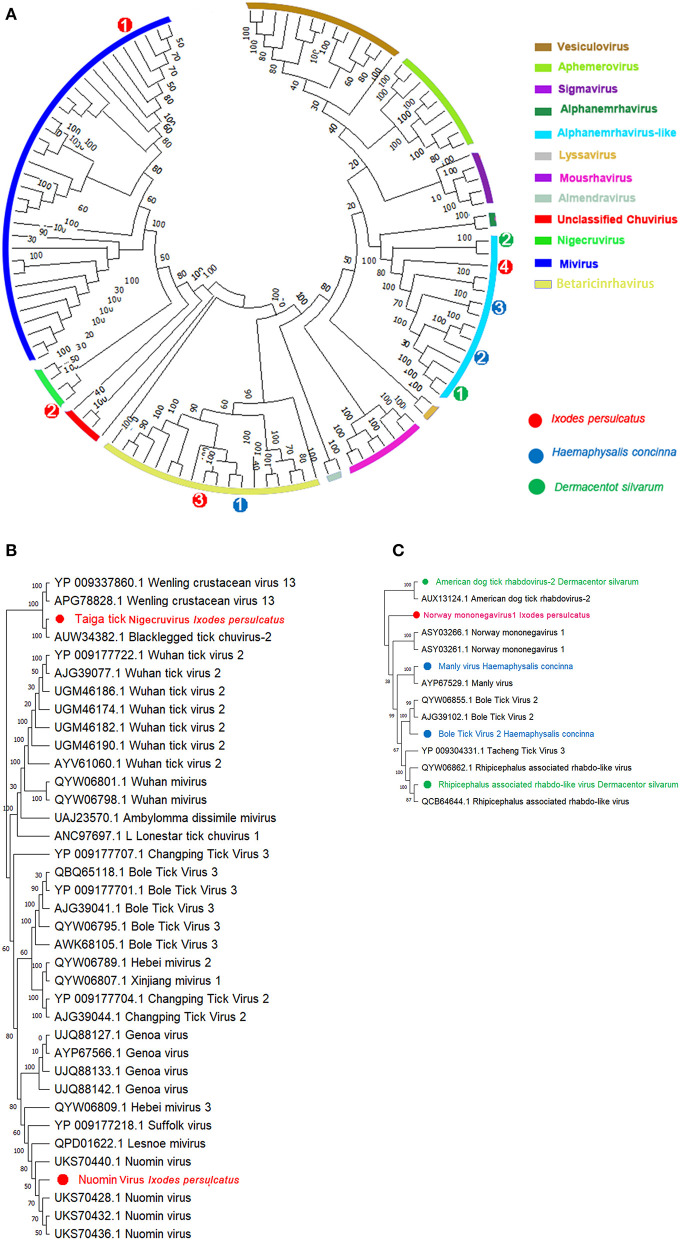
Phylogenetic analysis of representative branches in the order *Mononegavirales*. **(A)** Alignment of all branches belonging to the order *Mononegavirales*. **(B)** Enhanced region showing the genera relationship of *Mivirus* and *Nigecruvirus*. **(C)** Enhanced region showing the relationships of *Mononegavirus* and *Rhabdovirus*. Red dots marked with 1 to 4 represent Nuomin chuvirus, taiga tick *nigecruvirus*, Chimay rhabdovirus, and Norway *mononegavirus*-like virus 1 from *Ixodes persulcatus*, respectively. Green dots marked with 1 and 2 represent *Rhipicephalus*-associated rhabdo-like virus and America dog tick rhabdovirus from *Dermacentor silvarum*, respectively. Blue dots marked with 1 to 3 represents blacklegged tick rhabdovirus-1, Bole tick virus 2, and Manly virus from *Haemaphysalis concinna*, respectively. Maximum likelihood tree inferred using the best-fit model of amino acid substitution (LG + I + Γ + F for all alignments) with 1,000 bootstrap replicates.

Assembly of the viral contigs from *I. persulcatus* yields two viruses belonging to *Chuviridae* (Figures 2A, B). Among them, a *Mivirus* was discovered from *I. persulcatus* with shared viral RdRp amino acid (aa) sequences at 99.95% on the 100% query coverage of Nuomin virus (UKS70432.1) (Table 1). Nuomin virus was reported as a negative-strand circular RNA virus, whose genome at a length of 10,863 nucleotides, coding open reading frames (ORFs) of RNA-dependent RNA polymerase (RdRp), glycoprotein (G), nucleoprotein (NP), and VP4 protein (Supplementary Figure 11, left), shows high similarity to RdRp of Lesone mivirus (QPD01622.1) and Suffolk virus (YP 009177218.1) with distinct amino acids sequences on VP4. Nuomin virus had been recognized as pathogenic to humans in Tahe, Heilongjiang province, China (46), and then assigned to the recently proposed *Mivirus* genus of *Chuviridae* (21). Fortunately, we also achieved a novel *Nigecruvirus*, tentatively named Taiga tick nigecruvirus, which was shown at a length of 11,436 nucleotides and identical to blacklegged tick chuvirus 2 in L (AUW34382.1), G (AUW34383.1), NP (AUW34384.1), and VP4 (AUW34385.1) (Table 1, Supplementary Figure 11, right) based on the amino acids' similarities at 83.07, 79.94, 66.22, and 67.31%, respectively, over the 100% coverage. Homology searches revealed this virus to be very distant from genus *Mivirus*. The similarities of the four ORFs of the novel taiga tick nigecruvirus and Nuomin virus ensure the prevalence of these two *Chuviridae* members in *I. persulcatus* in China (47).

#### Mononegaviridae-like virus

Except for *Chuviridae*, we also identified several viral contigs belonging to *Mononegavirales* (48), among which one *Nucleorhabdovirus* and two *Alphanemrhavirus*-like viruses (Tacheng tick virus 3 and Norway mononegavirus 1) were discovered from *I. persulcatus* with 88.44, 47.32, and 49.46% identities to those aligned RdRp sequences of Chimay-like rhabdovirus (62.52% AVM86063.1), Tacheng tick virus 3 (YP_009304331.1), and Norway mononegavirus 1 (ASY03261.1) (Table 1) (Figures 2A, C, Supplementary Figure 5). Moreover, similar glycoprotein (G) and nucleoprotein (NP) sequences were also obtained from *I. persulcatus* and assigned as glycoprotein of Chimay rhabdovirus (AVM86062.1) and nucleoprotein of Tacheng tick virus 3 (62.17%, AJG39137.1) and Norway mononegavirus 1 (57.43%, KAG0427517.1), respectively (Table 1). As a case of *H. concinna*, assembly contigs obtained were shown with a high degree of diversity in *Mononegavirales* involving three *Alphanemrhavirus*-like rhabdoviruses and one *Nucleorhabdovirus*. Of *Alphanemrhavirus*-like rhabdovirus, Bole tick virus 2, Tacheng tick virus 3, and Tahe rhabdovirus 2 were involved, and their RdRp sequence accession numbers of reference strains as YP_009287864.1 (51.40%, 804 aa), YP_009304331.1 (48.29%, 2,179aa), and UXX19014.1 (99.69%, 2,168aa) in L segments, respectively (Table 1) (Figures 2A, C, Supplementary Figure 5). Bole tick virus 2 was also identified with nucleoprotein segment (73.02%, YP_009287860.1) over 100% coverage. The nucleorhabdovirus shared 73.53% similarities as (1,568aa) in RdRp genes of blacklegged tick rhabdovirus-1 (AUW34390.1), whereas *D. silvarum* from Zhaluntun, Inner Monogolia, yield two *Alphanemrhavirus*-like viruses identical to *Rhipicephalus* associated rhabdo-like virus (QYW06859.1) and American dog tick rhabdovirus (AUX13124.1) with 94.51% and 48.42% similarities over the 99.0% coverage, respectively (Figures 2A, C). The details of other related protein fragments in the viruses described earlier are shown in Table 1.

#### Bunyavirales

**Figure 3:**
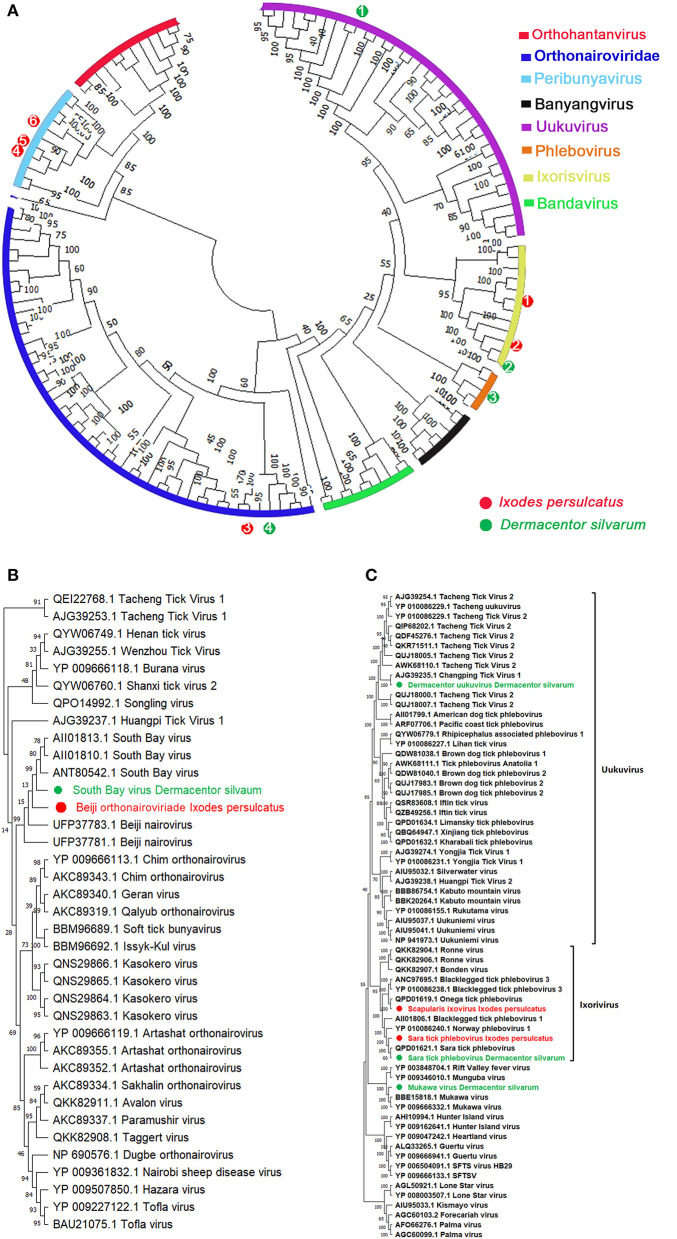
Phylogenetic analysis of representative branches in the order *Bunyavirales*. **(A)** Alignment of all branches belonging to the order *Bunyavirales*. **(B)** Enhanced region showing *Phenuiviridae* members. **(C)** Enhanced region showing *Orthonairoviridae* members. Red dots marked with 1 to 6 represent *Scapularis ixovirus*, Sara tick phlebovirus 1, Beiji *orthonairovirus*, unclassified *Bunyavirales* 1, unclassified *Bunyavirales* 2, and *Ixodes scapularis* orthobunyavirus viruses from *Ixodes persulcatus*, respectively. Green dots marked with 1 to 4 represent Tacheng uukuvirus, Sara tick phlebovirus 2, Mukawa virus, and South Bay virus from *Dermacentor silvarum*, respectively. Maximum likelihood tree inferred using the best-fit model of amino acid substitution (LG + I + Γ + F for all alignments) with 1,000 bootstrap replicates.

Bunyaviruses are segmented negative-stranded viruses that include at least nine families and 13 genera, many of which were involved as pathogens to humans and animals (49). In our study, 11 species in five genera of three families in the order *Bunyavirales* were detected. Assembly of these contigs revealed the presence of two *Orthonairovirus* (*Nairoviridae*), three *Phlebovirus*, two *Ixovirus*, and one *Uukuvirus* (*Phenuiviridae*), one *Orthobunyavirus* (*Peribunyaviridae*), and two unclassified Bunyaviruses from *I. persulcatus* and *D. silvarum*, whereas no *Bunyavirales* contigs detected in *H. concinna* (Table 1). The phylogenetic tree of *Bunyavirales* constructed based on the RdRp genes indicated that these phleboviruses, orthonairoviruses, and unclassified bunyaviruses clustered with referenced strains and formed well-supported monophyletic groups as the “classic” schematic diagram involved with *Orthohantanviridae, Nairoviridae, Phenuiviridae, Peribunyaviridae*, and others (Figure 3A).

#### Orthonairoviridae

Assembly of multiple contigs from *I. persulcatus* and *D. silvarum* pools contained with coding sequences is similar to viruses in the genus *Orthonairovirus* by BLASTx. With CCHFV as a reference genome, assembly of these *Orthonairovirus*-like contigs provided 100% coverage of L and S segments. The assembled sequences from *I. persulcatus* showed high identity (99.81%) to Beiji *orthonairovirus* (BJNV) (Table 1, Figures 3A, B), a novel species initially discovered from *I. persulcatus* (50) and human patients (51). BJNV reads were the dominant viral reads obtained from all *I. persulcatus* pools and accounted for 5–7% of filtered reads. Moreover, South Bay virus was also revealed from *D. silvarum* with 97.12% similarity in RdRp (AII01810.1) and 77.61% in nucleocapsid protein (AII01798.1) over the 100% coverage (Table 1, Figures 3A, B). However, despite an exhaustive bioinformatics analysis, we were unable to identify any contigs or reads with any similarity to M segments of *Orthonairovirus*.

#### Phenuiviridae

In the present study, a total of six species of three genera in the family *Phenuiviridae* are present in *I. persulcatus* and *D. silvarum*. The most diversity appeared in genus *Phlebovirus*, which is currently comprised of over 70 phleboviruses isolated from ticks, mosquitoes, and sand flies (52). We identified multiple contigs with homology to phleboviruses by BLASTx in pools of *I. persulcatus* and *D. silvarum* (Table 1, Figures 3A, C). The contigs derived from the pools of each tick species were assembled separately to a reference phlebovirus genome. We obtained phlebovirus contigs from *D. silvarum*, which were identical to Mukawa virus with shared amino acids similarities in L segments (98.60%, YP 009666332.1) and non-structural protein (93.53%, YP_009666334.1) and nucleoprotein (99.60%, YP_009666333.1) over the 98% coverage (Table 1, Figures 3A, C). Moreover, an unclassified *Phlebovirus*, Changping tick virus 2 (AJG39235.1), was harvested from *D. silvarum* with 85.46% similarity on the 100% sequence coverage of RdRp gene (Table 1, Figures 3A, B). In addition, two members in genus *Ixovirus* were also achieved, one is Sara tick phlebovirus from both *D. silvarum* and *I. persulcatus* with their RdRp identities up to 99.32 and 99.41%, respectively, across the 2,210 amino acids sequences (Table 1, Figures 3A, C). Another *Ixovirus* in *Phenuiviridae, Scapularis ixovirus* (previously cited as blacklegged tick Phlebovirus 3, YP_010086238.1), was also documented in *I. persulcatus* with 74.1% identity in L segments (Table 1, Figures 3A, C). Fortunately, the third genus *Uukuvirus* in the family *Phenuiviridae* was harvested from *D. silvarum* and identical to Tacheng *Uukuvirus* (Tacheng tick virus 2, AWK68110.1) with 92.61% similarity on the amino acids' sequences on polymerase gene (Table 1, Figures 3A, C). All sequences assigned to the *Phenuiviridae* family were presented with missed M segment coding for the viral glycoprotein.

### Other *bunyavirales*

Except for *Nairoviridae* and *Phenuiviridae*, we also obtained another viral contig belonging to *Bunyavirales* from *I. persulcatus* (Figure 3A, Supplementary Figure 6), which exhibited a relatively lower identity with Volzhskoe tick virus 1 (72.41%, QPD01626.1) (53) and *Ixodes scapularis* bunyavirus (38.25%, BBD75425.1) (*Orthobunyavirus, Peribunyaviridae*) based on over 90% sequence coverage of RdRp gene (Table 1). Moreover, Ubmeje-like virus (QKK82912.1) from *D. silvarum* was presented with a lower RdRp identity of 46.78%, which was also known as an unclassified *Bunyavirales* (Table 1).

### Double-stranded RNA viruses

Both *Partitiviridae* and *Totiviridae* are double-stranded RNA viruses mostly known as abundant and diverse in arthropods, some of which are pathogens to animals and humans. Notably, several lineages of *Partitivirus* appear related to endogenous virus elements in the genomes of these arthropod hosts (54). We identified three highly divergent *Partiti*-like viruses, Jilin partiti-like virus 1 from three *Ixodes persulcatus* pools (Table 1, Supplementary Figure 12). Interestingly, this virus formed a cluster with other arthropod-associated partiti-like viruses, which shared a close relationship with those found in host animals associated with arthropods. Whether Jilin partiti-like virus 1 is a truly tick-borne virus will need to be examined in detail. In addition to partiti-like virus, another dsRNA virus close to the family *Totiviridae* was also discovered from *H. concinna* with relatively lower amino acid identities with over 95% coverages of RdRp genes (50.01%, AUX13136.1) and ORF1 (67.01%, AUX13135.1) of Lonestar tick totivirus (Table 1). Meanwhile, *I. persulcatus* yields contigs similar to Hubei toti-like virus 24 based on over 95% sequences L segment (38.17%, YP_009336908.1) (Table 1). As a case of *D. silvarum*, both Lonestar tick totivirus and Hubei toti-like virus 24 were found with RdRp genes (Table 1, Supplementary Figure 13).

### Others

Other more distantly related viruses including unclassified Vovk virus (44.24% RdRp aa identity with QKK82915.1) and Levivirus (42.27% RdRp aa identity QDH88856.1, 31.75% H4bulk (QDH87337.1) and 35.34% coat (UJQ85258.1) in the family of *Leviviridae* were also detected from *I. persulcatus* (Table 1).

## Discussion

This study focused on the characterization of virome diversity of questing *D. silvarum, I. persulcatus*, and *H. concinna* ticks from North China. The outcome from our sampling efforts supports the results throughout various Chinese regions, showing that *I. persulcatus, D. silvarum*, and *H. concinna* are three of the most abundant questing ticks within the regions (55). All three tick species have been heavily implicated in the transmission of TBDs throughout the regions. *I. persulcatus* is the principal vector of the agents of Lyme borreliosis, Rickettsioses, and Babesioses along with *A. phagocytophilum* and clinically relevant human pathogen Jingmen tick virus (Alongshan virus) and Far-Eastern strain of TBEV (56). *D. silvarum* and *H. concinna* had also been linked with several tick-borne pathogens found throughout China, including *Francisella tularensis, Coxiella burnetii, Rickettsia* spp., *Babesia* spp., *Anaplasma* spp., TBEV, and SFTSV/Dabie bandavirus (50, 57). Despite examining more pools of *D. silvarum* and *H. concinna*, we identified a greater number of viral contigs in *I. persulcatus*, while no *Bunyavirales* viral sequences were detected within *H. concinna* pools. Combined, these studies support a hypothesis that different tick species can harbor diverse viruses at different levels. Interestingly, no viral sequence of TBEV was identified in the known endemic region, in addition to a low abundance of Jingmen tick virus identified. This most likely is attributed to the low prevalence of these viruses within the tick populations along with sampling bias. For example, data show that the prevalence of TBEV maintained within *I. persulcatus* populations in China is <0.1% (58). Since we only examined 90 *I. persulcatus* ticks, it is unlikely we would identify a positive tick.

### Diverse *Bunyavirus* predominated in our HTS data

More than 350 viruses are classified in the order *Bunyavirales*, which were assigned as nine families and over 13 genera, with various species only recently discovered and characterized (50). Our studies have demonstrated molecular evidence of the emergence of over 10 bunyaviruses present in ixodid ticks in China. Based on the phylogenetic analysis of the polymerase coding regions, Beiji *orthonairovirus* and South Bay virus form a distinct phylogenetic lineage within currently described orthonairoviruses, which indicate that the genus *Orthonairovirus* is significantly more diverse than previously appreciated. Based on a very short sequence fragment, the earlier phylogenetic analysis of tick-associated *orthonairoviruses* yielded two main phylogenetic clades, with which one clade being isolated exclusively from ixodid ticks and the other from argasid ticks (59). The phylogeny has been proven complex and confounded as some previously unidentified genetically distinct or emerged *Nairovirus* presents. A more accurate *Orthonairovirus* classification scheme is urgent to be updated with oncoming contributions from genome sequencing of unclassified *Nairovirus*, especially those isolated from ticks of Argasid or Ixodid. In addition to the genus *Orthonairovirus*, we also identified several *Phenuiviridae* viruses from *I. persulcatus* and *D. silvarum*. *Phenuiviridae* was the latest update representing the newly combined *Phlebovirus* and *Tenuivirus* along with the newly erected genera *Goukovirus* and *Phasivirus* (49). Historically, phleboviruses were classified into at least three phylogenetic clusters, each comprised of several potential species: the Uukuniemi group, the Bhanja group, and the STFS group based on vector, genomic, and serological relationships (49). Of them, Tacheng uukuvirus in *D. reticulatus* has been proven phylogenetically close to a human clinical isolate with a history of tick bite in northwestern China (60), which raised widespread concerns about the health risk of *Dermacetor* ticks to transmit *Uukuvirus* in China. As one of the predominant human-bitten species, the potential transmission ability of *Uukuvirus* in *D. silvarum* remains to be determined in detail. Furthermore, the Mukawa virus, another novel *Phlebovirus* outsides the described three clades earlier, was first detected from *D. silvarum*. Together with the evidence of Mukawa virus proliferation in the Huh-7 cell line and the innate immune responses caused by the viral NSs during mice infection (61), the immense medical significance of *D. silvarum* to human victims should be addressed and more detailed surveys on the epidemiological characters of Mukawa virus should not be ignored (62). Similarly, although no clinical manifestations were documented to elucidate the pathogenicity of some phleboviruses in humans and animals, the discovery of Sara tick phlebovirus, *Scapularis ixovirus*, and Changping tick virus 2 in our studies has deepened our knowledge about diverse *Phenuiviridae* viruses widely prevalent in China and adjacent areas. However, the relative abundance of these *Bunyavirales* viruses was shown as quite lower among the viral transcripts obtained. The expansive infectious ticks, for example, up to 3% infection rate of Beiji *orthonairovirus* in *I. persulcatus* (9), might cause a series of human victims suffering from bunyavirus infections. Considering the unknown pathogenicity of Volzhskoe tick virus, Ubmeje virus, and *Ixodes scapularis*-orthobunyavirus discovered in the present article, the findings of *Bunyavirus*-like contigs from *I. persulcatus* and *D. silvarum* suggest that these viruses may have much medical relevance and broader geographical distributions, perhaps throughout the range of the two tick species, comparable to bacterial and protozoan pathogens transmitted by them. Enhanced by the spreading of ticks through the movements of the viremic host reservoir, the real geographical ranges should be further extended. We do not know whether these bunyaviruses are specific to their vector tick species. As a matter of fact, some tick-borne viruses can usually be isolated from more than one tick species. CCHFV, for example, has been isolated from over 30 tick species and multiple genera throughout Europe, Africa, and Asia, and it is unlikely that all of the 30 species represent competent vectors (16, 63). Although the HTS analysis suggests vector specificity for tick species, we cannot rule out the possibility that other tick species may serve as vectors of these viruses.

One particularly notable characteristic of these Bunyaviruses is the lack of a recognizable glycoprotein-coding segment. Although we recovered ~90% of the L and S segments for Sara tick phlebovirus, Mukava virus, and other Bunyavirus by HTS and dispelled the possibility of viral integration, we were unable to identify any scenario with similarity to *Bunyavirales* M segments. We have considered various explanations for this confounding result. First, these viruses encode a more complicated secondary structure of the M segments, which may inhibit efficient cDNA synthesis, subsequently resulting in the failure to detect by amplifications (23). Alternatively, these viruses may employ special pathways in the process of cellular attachment and entry other than the well-known glycoprotein precursor (GPC) depending manner (64). Finally, we also cannot exclude the possibility that an episome-like form is coded by S and L segments of these viruses. As such, they may not assemble an infectious virion but instead use other vehicles, for instance, extracellular vesicles, for their dissemination to new hosts (65).

### High diversity *Mononegavirales* contigs predominated in our HTS data

In our analysis, a novel *Chuvirus*, Taiga tick nigecruvirus, was also revealed in *I. persulcatus* other than Nuomin virus. Phylogenic analysis based on L, G, NP, and VP4 fragments suggested that Taiga tick nigecruvirus and blacklegged tick chuvirus 2 formed a separate monophyletic clade outside genus *Mivirus* under the *Chuviridae* branch of *Jingchuvirales* in the order *Mononegavirales*, although a complete genetic characterization will be required to fully determine its taxonomy. The taiga tick nigecruvirus was found to have high identity in RdRp and relatively lower identities in N and G genes with blacklegged tick chuvirus 2, with no pathogenicity in humans and other animals. However, the reported human pathogenicity of Nuomin virus in genus *Mivirus* from Tahe, Heilongjiang province, suggested the potential threats of the Taiga tick nigecruvirus on humans and animals *via* tick bites or other transmission routes. Based on the phylogeny relationship, we considered the virus closely related to blacklegged tick chuvirus 2 as a novel Chuvirus named Taiga tick nigecruvirus tentative, while Nuomin virus as a member of *Mivirus*, called *Mivirus nuomin*.

Rhabdoviridae are another diverse set of single-stranded negative-sense RNA viruses with frequent host-switching features during their evolution history (66) and widely found to naturally infect both animals and plants. In the present study, almost all the pools of the three tick species yield the positive contains of *Rhabdovirus*-like contigs, which demonstrates relatively high divergence of RdRp sequences of the rhabdoviruses. Among these rhabdoviruses, only two *Nucleorhabdovirus*, Chimay-like rhabdovirus and blacklegged tick rhabdovirus-1, were harvested from *I. persulcatus* and *H. concinna*, respectively. While, in the *Alphanemrha* virus-like group, extensive diversity was observed with three virus species in *H. concinna*, two species in *D. silvarum*, and one species in *I. persulcatus*. Together with two *Nucleorhabdovirus* detected, diverse *Rhabdovirus* in the three tick species might be associated with high frequencies of host switches within their comparable broader host spectrums. It should be noted that more diversity of *Rhabdovirus* is still unexplored due to limited tick samples or geographical sites.

Although our surveys have demonstrated a wide array of highly diverse viruses harbored in the three tick species sampled, however, the limitations of the study were also prominent due to our sample bias. One constraint of our study was caused by the limited geographical regions and aggregated distributions of these ticks, as our HTS analysis merely focused on specimens collected from limited locations. We could not determine the pathogenicity and transmission capability of the viruses carried by these ticks. Necessary investigations should be carried out in detail to characterize their epidemiological features and determine their potential threats to public health. Meanwhile, in our sequence-based query strategies, the presence of “dark contigs” which had no BLAST hits, may include novel, highly divergent viruses that are unrecognizable (Supplementary Table 1). We still anticipate that many of the viruses uncovered by our HTS analysis would be along the distribution ranges of their presumed natural cycle between vector ticks and host animals, and we also speculate that analysis of ticks from diverse geographical areas would reveal greater viral diversity with more emerged agents to be discovered in future. Thus, accurate viral taxonomy would allow more precise elucidation of tick-associated virus evolutionary relationships (67).

## Conclusion

In summary, we metagenomically analyzed the virome of *I. persulcatus, D. silvarum*, and *H. concinna*, three predominant tick species associated with pathogens transmitted in China. In addition to the exploration of viral diversity, our study also identified several tick-borne viruses with potential relevance for diseases of humans and livestock, for example, Tacheng uukuvirus, Sara tick-borne phlebovirus, Beiji *orthonairovirus*, Nuomin virus, and Jingmen tick virus. The novel viruses found in the present study, such as *Dermacentor* pestivirus-like virus, taiga tick nigecruvirus, Mukawa virus, South Bay virus, and *Scapularis ixovirus*, do not allow reliable conclusions on the transmissibility issues merely depending on available genetic similarities to human pathogens. Our discoveries have paved a cornerstone for more detailed investigations and rational control strategies for tick-borne viral diseases in China.

## Data availability statement

The datasets presented in this study can be found in online repositories. The names of the repository/repositories and accession number(s) can be found in the article/Supplementary material.

## Author contributions

YS contributed to the concept, design, and statistical analysis of the manuscript. TQ and MS were involved in the field survey. YS and HF were involved in the taxonomic identification of ticks. TQ and ZL were involved in the sequencing and molecular analysis of viruses. YS, TQ, MS, and MZ were involved in the analysis, interpretation, and drafting of the manuscript. All authors contributed to revising it critically for important intellectual content, final approval of the version to be published, and agreement to be accountable for all aspects of the manuscript in ensuring that questions related to the accuracy or integrity of any part of the manuscript are appropriately investigated and resolved.
