# Supplementary material for: Diversity of RNA viruses of three dominant tick species in North China
Source: Front Vet Sci. 2023 Jan 13;9:1057977. doi: 10.3389/fvets.2022.1057977 (PMC9880493; doi:10.3389/fvets.2022.1057977)
Supplement: Supplementary file 1 [file Data_Sheet_1.docx]

Supplementary Material

# Supplementary Figures and Tables

For more information on Supplementary Material and for details on the different file types accepted, please see [here](http://home.frontiersin.org/about/author-guidelines#SupplementaryMaterial). Figures, tables, and images will be published under a Creative Commons CC-BY licence and permission must be obtained for use of copyrighted material from other sources (including re-published/adapted/modified/partial figures and images from the internet). It is the responsibility of the authors to acquire the licenses, to follow any citation instructions requested by third-party rights holders, and cover any supplementary charges.

## Supplementary Tables

**Supplemental table S1. Metagenome data obtained from 8 pools of three dominant tick species in China**

*****dark contigs which had no BLAST hits, may include novel, highly divergent viruses that are unrecognizable.

**Supplemental table S2. Primer pairs used for PCR in molecular investigation of viruses^#^**

| **Virus** |  | **Sequences** | **Size** |
| --- | --- | --- | --- |
| Taiga tick nigecruvirus | F1 | 5'-TCACTGAGCAGGAATACTTTG-3' | 530 |
|  | R1 | 5'-CCTTAGATCGGACGACATAC-3' |  |
|  | F1in | 5'-TGAGCAGGAATACTTTGAT-3' | 480 |
|  | R1in | 5'-AATACTCCCTTGGACAGC-3' |  |
| Nuomin virus | F1 | 5'-VAAGGCAGGGGAAAGAAAGAAGATGG-3' | 1450 |
|  | R1 | 5'-CAGCATCAGGCACATAAACAAAAGA-3' |  |
|  | F1in | 5'-GAACTGTTATTGGAAATGCGGAGAG-3' | 1287 |
|  | R1in | 5'-CAGCATCAGGCACATAAACAAAAGA-3' |  |
| BLTV4 | F1 | 5'-CACTCTGTATGCGACTGTTTGA-3' | 521 |
|  | R1 | 5'-GCAGGTCCTCACGAAAGCAG-3' |  |
|  | F1in | AGCTTCTATGACAAAGAGCAC-3' | 487 |
|  | R1in | 5'-ATTCAGGAGCAGGTCAGAAAC-3' |  |
| JMTV | F1 | 5'-GCTCAAGACCACTGTAGCGT-3' | 550 |
|  | R1 | 5'-GCACTACACCCAATCACGGA-3' |  |
|  | F1in | 5'-TACGTGTCGGCGTACTGTTT-3' | 480 |
|  | R1in | 5'-CGGAGTACGTTAGGCAGCTC-3' |  |
| pan-phlebovirus* | F1 | 5'-GGCTACTTCAARAAYAARGANGA-3' | 507 |
|  | R1 | 5'-CTCTCTCAGICCICCRTGYTG-3' |  |
| Uukuniemi virus | 2.8 kF | 5′-TTC AAR AAR CMT CAR CAT GG-3′ | 500 |
|  | 3.3kR | 5′-TGC AGK ATK CCY TGC ATC AT-3′ |  |
| Mukawa virus | 2759F | 5'-CAGCATGGIGGIYTIAGRGAAATYTATGT-3' | 510 |
|  | 3276R | 5'-GAWGTRWARTGCAGGATICCYTGCATCAT-3' |  |
| Sara tick phlebovirus | F1 | 5'-CCATCAATCTGTACACCAGG-3' | 895 |
|  | R1 | 5'-ACACAAAGTCCGCCCATTAC-3' |  |
| Beiji orthonariovirus | P1 | 5'-TTGGTGATATTGATGAAAGCAAGAGTGAG-3' | 431 |
|  | P2 | 5'-RTCAAACACYCGTAGTGGTCCAGGAAC-3' |  |

* The primer sets were utilized to support clues for potential phlebovirus.

#Nonstandard nucleotides are as follows; I, inosine; R, adenine (A) and guanine (G); S, G and cytosine (C); W, A and thymine (T); Y, T and C.

**Supplemental Table S3.** Primer used for amplifying the complete genome of Taiga tick nigecruvirus (TTNV) by nested RT-PCR with overlapping primers, genome walking and rapid amplification of complementary DNA ends (RACE).

| **Primer function** | **Forward (5'→3')** | **Reverse (5'→3’)** | **Position** |
| --- | --- | --- | --- |
| 5' RACE | RSP1: CTAATAAGCCTCACTATAGGGCAAGCAGTGGTATCACAGCAGA | 5’RACE-L1: GTCAACCAGCCCAACACTCCACA | P1: 1-517 |
|  | RSP2: TCAATACGACTCACTATAGCGG | 5’RACE-L1-1: GTCTGGTCGGTTGTGGTATGTGTTTG |  |
| Genome walking | AP1^*^ | CV-L2-1: AGCACTCGCCACATAGTCATCC | P2: 424-2120 |
|  | AP2^*^ | CV-L2-2: AAGTAACATTCAGATGCGTGGA |  |
|  | AP3^*^ | CV-L2-3: TCACCGCTATCCCTACTACATA |  |
|  | AP1^*^ | CV-L3-1: GGTCACAATCTGCGGTTACT | P3: 1883-3114 |
|  | AP2^*^ | CV-L3-2: GTTGTAGGCGTCATGGTCGCT |  |
|  | AP3^*^ | CV-L3-3: TCTTGTTGTCGGCATATT |  |
|  | AP1^*^ | CV-L4-1: ATTATGAGGGCGATGCGT | P4: 2983-4523 |
|  | AP2^*^ | CV-L4-2: TCTTCCTCGCCGCTCTC |  |
|  | AP3^*^ | CV-L4-3: GCAACAGGCGATTCCGAAACG |  |
|  | AP1^*^ | CV-L5-1: GCTTCGTTCTTCCTCGCCACAT | P5: 4162-5900 |
|  | AP2^*^ | CV-L5-2: ATCCCACTGGCGTCATACCTCT |  |
|  | AP3^*^ | CV-L5-3: GTGCCCTCATCAGCGACCACG |  |
|  | AP1^*^ | CV-L6-1: AGCTCGCCCTCATACACTAC | P6: 5824-7053 |
|  | AP2^*^ | CV-L6-2: TCTGCGTACTCAGGGTCAATA |  |
|  | AP3^*^ | CV-L6-3: GCACTTTCAGCACTTCATTTGTCA |  |
| Nested RT-PCR | CV-U7: CCTTGATTACTCTCGTTCATACT | CV-L7: GCACTTTCAGCACTTCATTTA | P7: 6719-7469 |
|  | CV-U7-1: CGTTACGCTTCGTTCTTCTC |  |  |
|  | CV-U8: ATTACGGATTTGACCCGAGT | CV-L8: TCCCCGTAGCCATGTATGTC | P8: 7425-7849 |
|  |  | CV-L8-1: TGTAGGTGACGGGAATTTCTG |  |
|  | CV-U9: CTCCATTTCCAGCTCAACTG | CV-L9: TTTACCGAGAATGTCCACCT | P9: 7644-8297 |
|  |  | CV-L9-1: ATGGTCAAGTGCGCTCCATG |  |
|  | CV-U10: CGGCTGTGGTGGACATTCTG | CV-L10: TATCGGGCGTAGTGCGTGAT | P10: 8125-10023 |
|  |  | CV-L10-1: GGCAATGAGTTCATCGATGA |  |
| 3’RACE | 3’RACE-U11: AACGAAGGATGGCATTTGGCGGC | GSP1: TCAATACGACTCACTATAGGGCAAGCAGTGGTATCAACGCAAG | P11: 9927-11435 |
|  | 3’RACE-U11-1: CGGGAGATCGGTGGTGGTAAAAG | GSP2: CTAATACGACTCACTATAGGGC |  |

*AP1~AP3 primers are provided by Genome Walking Kit (TAKARA, Code No. 6108).

**Table S4.** Primer used for amplifying L gene of Mukawa virus (MKWV) by nested RT-PCR with overlapping primers and rapid amplification of complementary DNA ends (RACE)

| **Gene** | **Forward (location) (5'→ 3')** | **Reverse (location) (5'→3 ')** |
| --- | --- | --- |
| **L** | L-5’Race-RSP1: CTAATACGACTCACTATAGGGCAAGCAGTGGTATCAACGCAGA | L-5’Race-GSP1: CTCAAAGTTCACTCCCATCTG |
|  | L-5’Race-RSP2: CTAATACGACTCACTATAGGGC | L-5’Race-GSP2 CCATCTGTAGTGAAAGTAGGGC |
|  | LF726: CTGCGCTACCATCCTTTC | L1823R2: CCTTATATTTCTTTGCCTCCAC |
|  | LF1715: CAGTATCACAGAACAACC | LR2065: CAAACCCTTCCATCATCA |
|  | LF2013: ATGTGATGATGGAAGGGT | LR3209: GTGCCAGATGTCCAGATT |
|  | LF3187: TYGCAGATGATGCTAGGAAGT | LR3894: TTGAAGCCAGGAGATTGTCTG |
|  | LF3821: AGAGGTGGAGACTTTGATT | LR4765: ATGAGAAGCAAGAGGGGA |
|  | LF4719: TCAGTGATAAGTGGTTCC | LR5844: TATCTGTTTGTTGAATCTCT |
|  | LF5732: GTCATCAGAGCAGTCAGTA | LR6653: CCACAAAACCATCCATCA |
|  | L-3’Race-RSP1: GTTCACTCCTTCACATGTTCCG | L-3’Race-GSP1: CTAATACGACTCACTATAGGGCAAGCAGTGGTATCAACGCAGA |
|  | L-3’Race-RSP2: CGTCTACTCATTGGGTGTCCT | L-3’Race-GSP2: CTAATACGACTCACTATAGGGC |

## Supplemental Figures


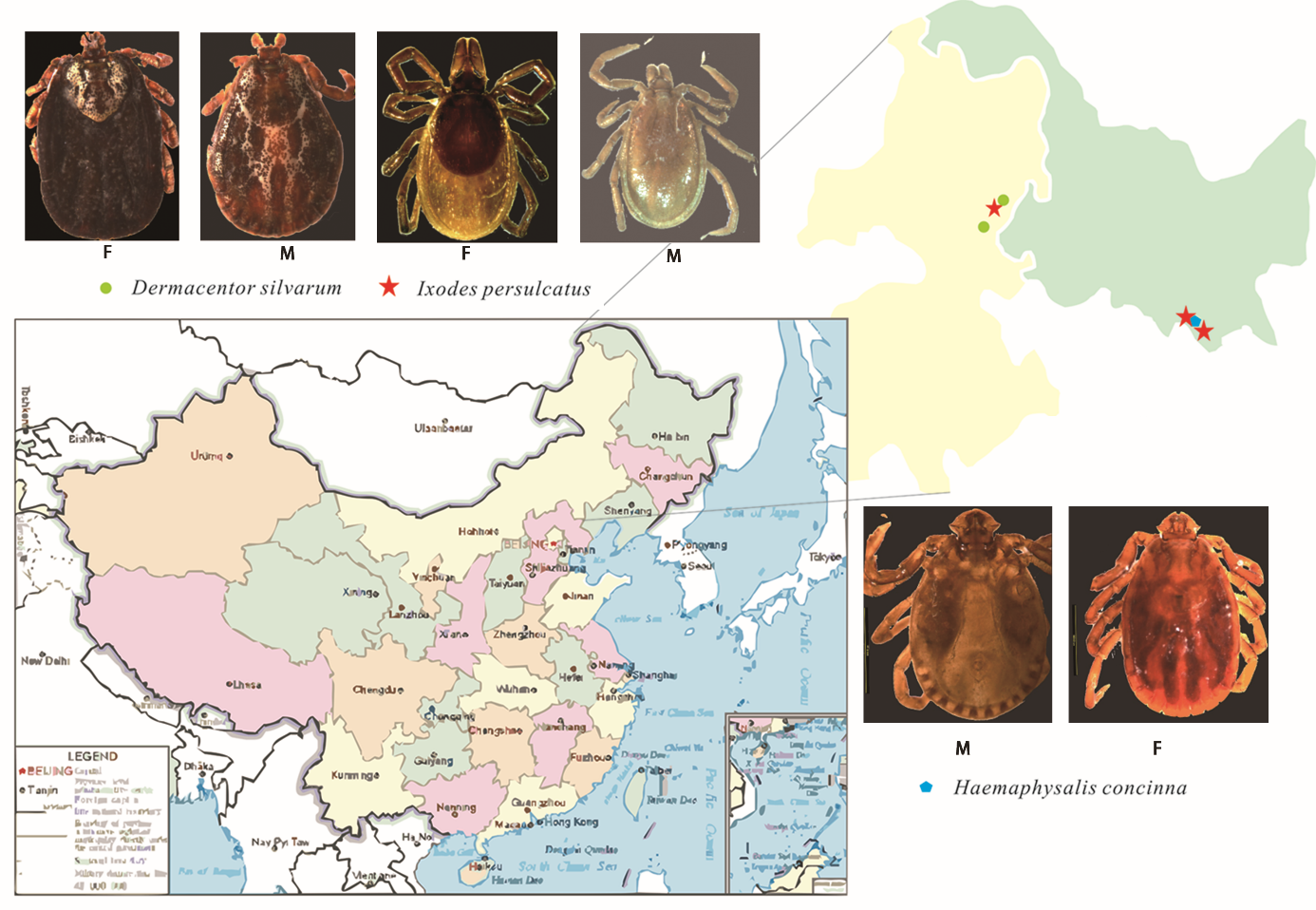


**Supplementary Figure S1.** **The origin sites of ticks sampled in the present study.**

(F: female; M male)

Red stars, *Ixodes persulcatus*. Blue pentagons, *Haemaphysalis concinna*. Green circles, *Dermacentor silvarum*.
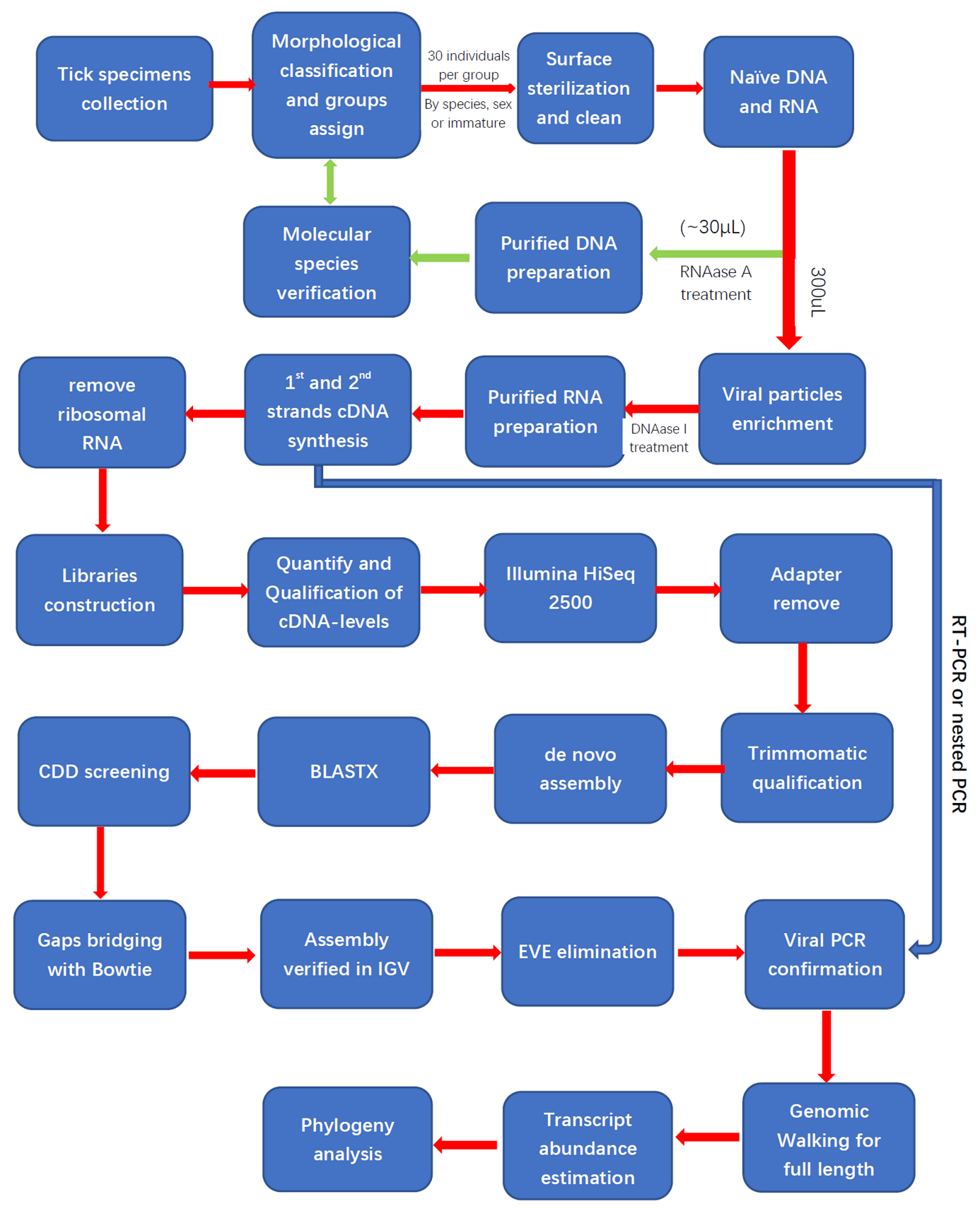


**Supplementary Figure S2. Study design diagram**

The flow chart demonstrates different types of treatment and analysis performed to investigate the presence of diverse viral polymerase in three tick species.





**Supplementary Figure S3.** **5’- and 3’-RACE amplification of** **Taiga tick nigecruvirus cDNAs using total RNA as a template for cDNA synthesis.**

Lanes 1 and 4: control RACE reactions primed with RSP1 or GSP1 alone. Lanes 2 518bp 5’-RACEfragment generated with 5’RACE-L1 and RSP1 primers. Lanes 3: 1508 bp 3’-RACE fragment amplified with 3’RACE-U11 and GSP1 primers. Lane M: DNA size marker





**Supplementary Figure S4**. **5’- and 3’-RACE amplification of Mukawa virus cDNAs using total RNA as a template for cDNA synthesis.**

Lanes 1 and 4: control RACE reactions primed with RSP1 or GSP1 alone. Lanes 2 728bp 5’-RACEfragment generated with 5’RACE- RSP1 and 5’RACE- GSP1 primers. Lanes 3: 716 bp 3’-RACE fragment amplified with 3’RACE- RSP2 and 3’RACE- GSP2 primers. Lane M: DNA size marker


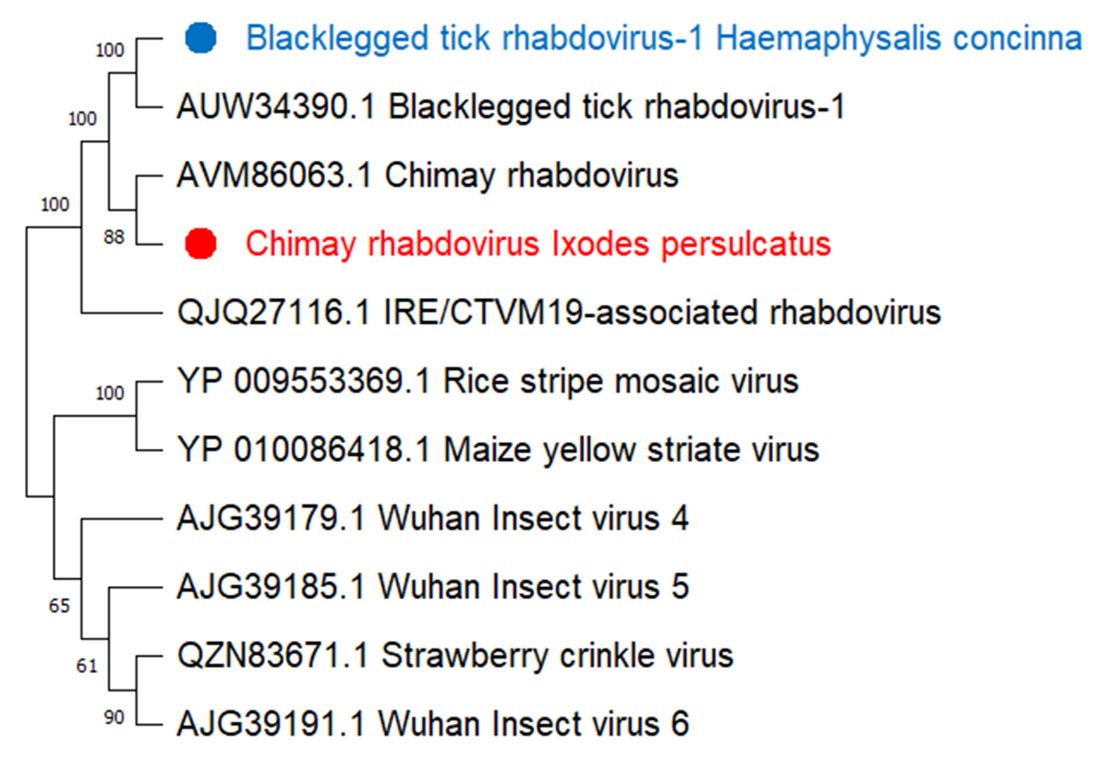


**Supplementary Figure S5. Phylogeny relationships in *Betaricinrhavirus*.**

Red balls: Viruses derived from *Ixodes persulcatus*

Blue balls: Viruses derived from *Haemaphysalis concinna*


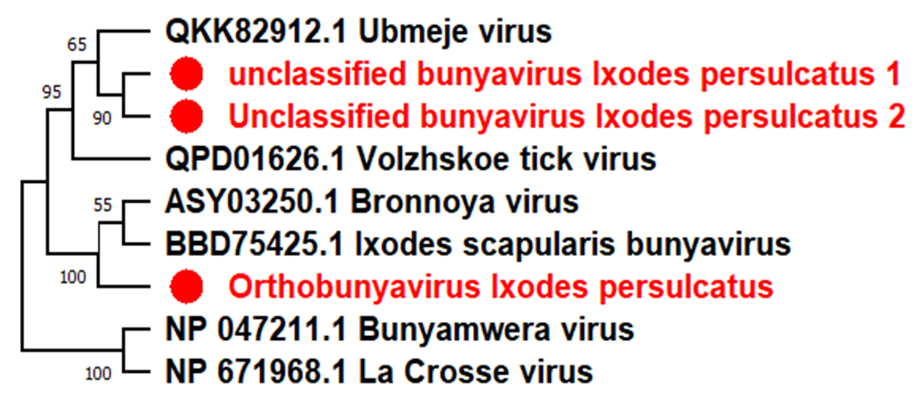


**Supplementary Figure S6. Phylogeny relationships in *Peribunyaviridae* members.** Red balls Viruses derived from *Ixodes persulcatus*


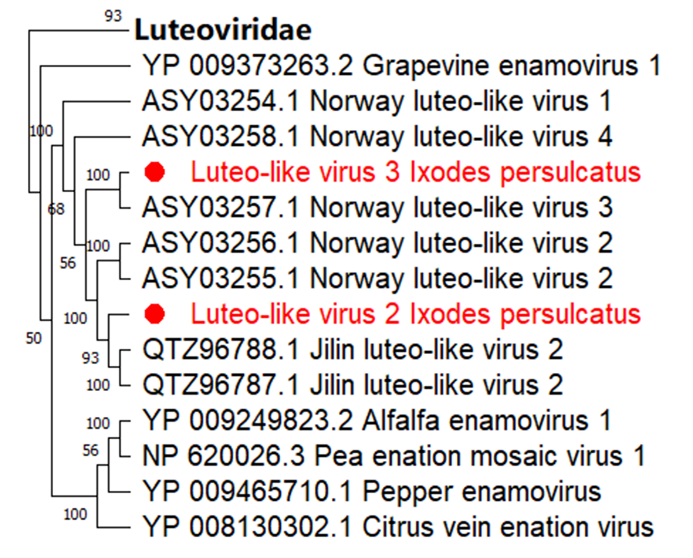


**Supplemental Figure S7. Phylogenetic analysis of representative branches in *Luteoviridae***.

Red balls, viruses from *Ixodes persulcatus*. Maximum likelihood tree inferred using the best-fit model of amino acid substitution (LG + I + Γ + F for all alignments) with 1000 bootstrap replicates.


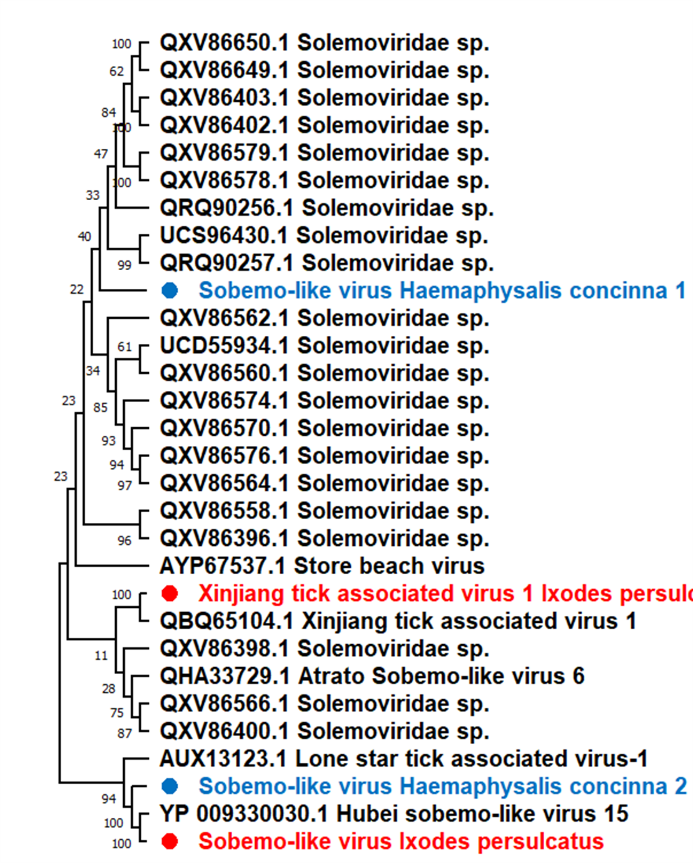


**Supplemental Figure S8. Phylogenetic analysis of representative branches in *Solemovirales*** Red balls, viruses from *Ixodes persulcatus*. Blue balls, viruses from *Haemaphysalis concinna*. Maximum likelihood tree inferred using the best-fit model of amino acid substitution (LG + I + Γ + F for all alignments) with 1000 bootstrap.


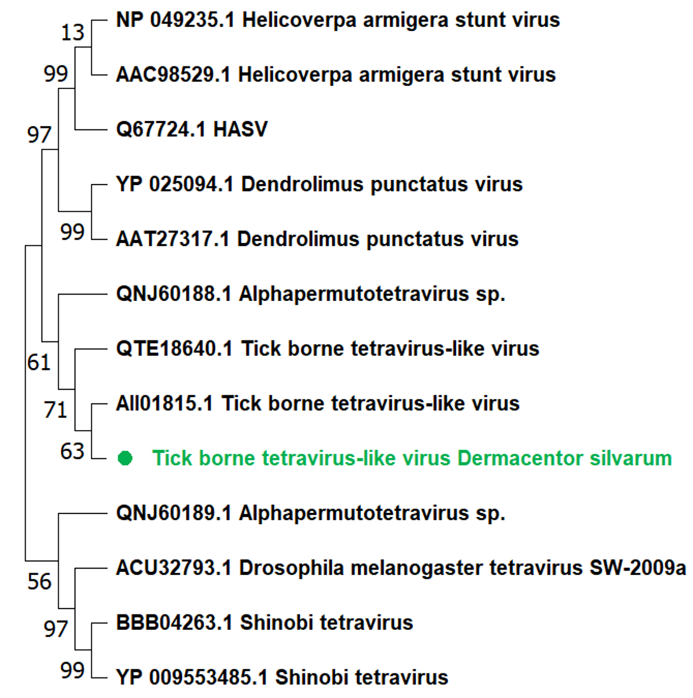


**Supplemental Figure S9. Phylogenetic analysis of representative branches in *Tetraviridae*** Green balls, virus from *Dermacentor silvarum*; Maximum likelihood tree inferred using the best-fit model of amino acid substitution (LG + I + Γ + F for all alignments) with 1000 bootstrap replicates.

**
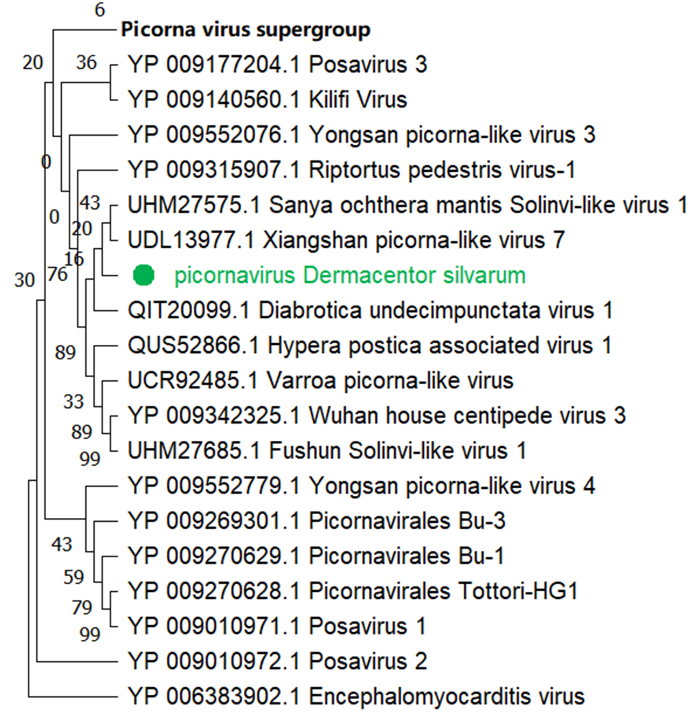
**

**Supplemental Figure S10. Phylogenetic analysis of representative branches in *Picoranviridae***

Green balls, virus from *Dermacentor silvarum*; Maximum likelihood tree inferred using the best-fit model of amino acid substitution (LG + I + Γ + F for all alignments) with 1000 bootstrap replicates.


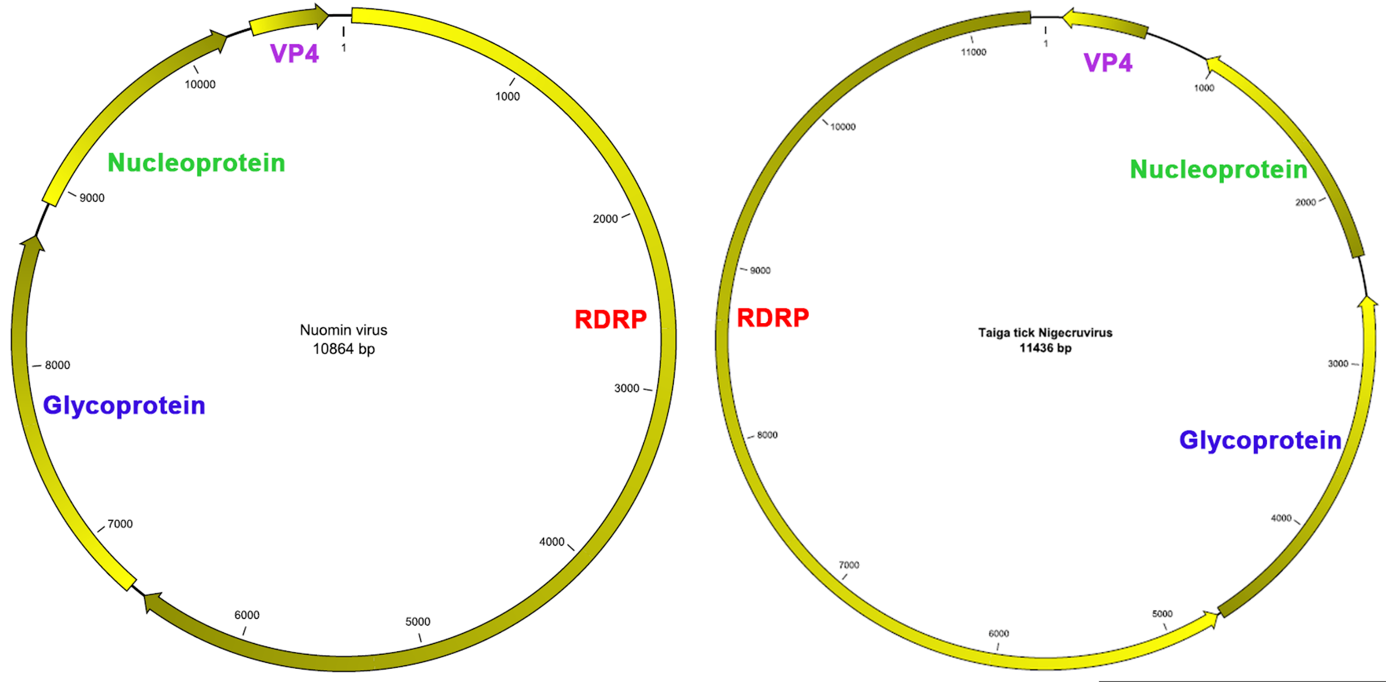


**Supplemental Figure S11. Genomic components of Nuomin virus (left) and Taiga tick nigecruvirus (right).**


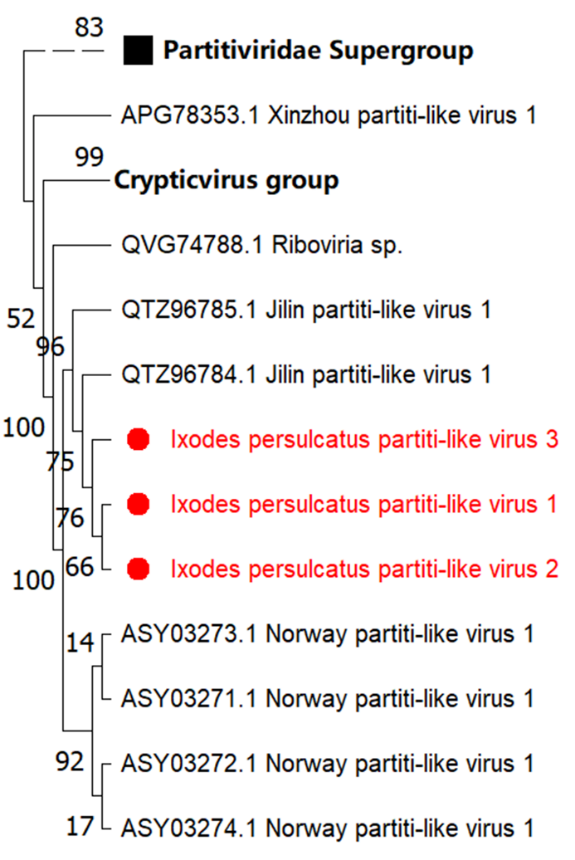


**Supplemental Figure S12. Phylogenetic analysis of representative branches in family *Partitiviridae***

Red balls, viruses from *Ixodes persulcatus*. Maximum likelihood tree inferred using the best-fit model of amino acid substitution (LG + I + Γ + F for all alignments) with 1000 bootstrap replicates.


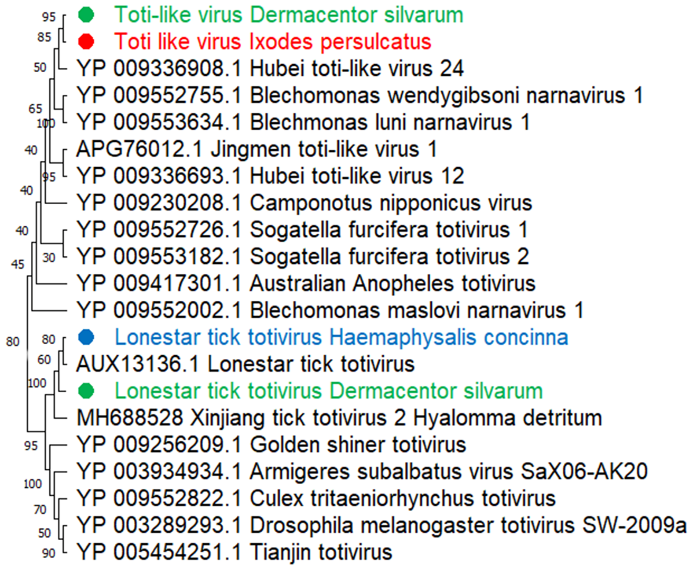


**Supplemental Figure S13. Phylogenetic analysis of representative branches in family *Toitiviridae***

Red ball, viruses from *Ixodes persulcatus*. Green balls, viruses from *Dermacentor silvarum*. Blue ball, virus from *Haemaphysalis concinna*. Maximum likelihood tree inferred using the best-fit model of amino acid substitution (LG + I + Γ + F for all alignments) with 1000 bootstrap replicates.
